# Bile and Digestion

**Published:** 1882-09

**Authors:** J. Wm. Stokes

**Affiliations:** Corinth, Miss.


BILE AND DIGESTION. By J. WM. STOKES, M. D., Corinth, Miss.

The investigation of the function of bile in its relation to digestion, involves the consideration of two dependent questions: I. Does bile perform *any* function in digestion? and II. What is that function? And since upon the answer rendered to the former depends the pertinence of the latter, we shall proceed directly to its consideration.

I. First, then, does bile enter at all as a factor into the process we call digestion?

When we consider that bile is the constant secretion of the largest gland in the body; that this gland is supplied with blood from two distinct sources, and in weight is nearly 3 per cent. of the whole body; the inference by analogy alone in the correlation of nature's processes, and especially those exhibited in the animal economy, is that it should perform some highly important function or functions in that economy. That it is poured into the upper part of the intestine when important processes of digestion are transpiring, and only traces of it find exit through the anus with the débris of nutrition, creates strong presumption of some office peculiar to it in digestion. It is still conceivable and possible, however, that bile is solely excrementitious in character; and hence we will first direct attention to this phase of the subject.

Now, bile is either (1) purely an excrement, or (2) enters as a factor in digestion—including in the term absorption. These two reciprocal propositions cover all the logical possibilities in reference to the presence of bile in the intestine; for if bile perform *any* function in digestion, it cannot be solely excrementatious in character, and *vice versa*. Of course, it might perform its function, be broken up in the process, and all its constituents discharged with the débris of the system; but even in that case it could not strictly be termed an excrement. Yet, not even in this acceptation would the term apply to bile. Hence we shall address ourselves in the first instance to the elimination of the possibility that bile is an excrement, and therefrom the last term in the above antitithesis will follow by necessity.

(1) Bile is not an excrement. Were this its character in reality, we should find it all, or its constituents, in the excretions of the body; and there are but two main routes by which it may find exit—(a) with the alvine dejections, or (b) with the urine. Hence it must all be passed with the fæces or with the urine, or complementary portions by each of these routes. No account is made here of the excretory functions of lungs and skin: since the composition of biliary matters is such as to preclude the possibility of their ejection from the system in this way.

(a) It is not all passed with the fæces; nor yet more of any element, than might be accounted for by accidental entanglement. As a matter of course, cholesterine, pre-existing in the blood as an excrementitious principle, is to be regarded as holding a *merely* accidental association with bile in common with other liquids and secretions of the body. With this preface, we proceed to scrutinize and compare the data upon the subject.

By careful and protracted observation, Bidder and Schmidt found that a dog weighing 8 kilogrammes excreted in five days 97.3 grm. fæces, 40.9 grm. of which were solid. The solid matters of bile secreted in the same time should be 39.52 grm.—i. e., nearly equal to the solid portion of the fæces in this case. But the fæces showed only traces of bile solids upon application of the usual tests; and in any case it is evident that vastly more than 1.38 grm. of the solid fæces must have been furnished in the given time, by the débris of nutrition and waste.

Now it is true, that if the bile solids had split up in their passage down the alimentary canal their elements would not give the characteristic reaction. How is it, then, in reference to the elements of bile in the fæces?

Sulphur is one of the most constant of these elements, and in bile of dogs amounts to 6 per cent. of the solids. Being very little subject to change of any kind, this element should be readily detected. Hence in 39.52 grm. solid bile secreted in 5 days, there should be 2.37 grm. sulphur. The fæces in the given case contained, however, only .334 grm. sulphur, more than half of which, it was estimated, must have been due to the abundant hairs present in the fæces, It is evident, therefore, that but a small proportion of the sulphur secreted in bile in the given time passed out with the fæces.

Cholalic acid also enters largely as an ultimate element of bile; and its presence is readily detected. In 1862, Hoppe collected for three days, and weighed, the fæces of an 8 kilogramme dog. They aggregated 287 grm.; in which was found by analysis 1.1 grm. cholalic acid for the entire time, or .36 grm. per day. Now, according to the accepted standard for a dog of that weight, the solid acids of bile secreted in 24 hours would be 4 grm. But .36 grm. cholalic acid, by synthesis, would represent .46 grm. tauro—or .41 grm. glyco-cholic acid; hence less than ½ grm. out of the 4 grm. secreted and discharged into the intestine, found exit with the fæces. It follows, therefore, that but a small portion of bile solids, and this portion mechanically entangled most probably with the débris of the system, is carried out with the fæces.

To this conclusion it may be objected that the premises are insufficient in number to establish their generality. It must be remembered, however, that the above are typical cases, expressing the *general results* of several experiments of several experimenters, and are accepted by physiologists as facts.

(b) We come now to consider the question in reference to the only other avenue of escape as an excrement—the urine. Unless investigation here gives evidence that the complementary portion of bile solids finds exit in this way, our proposition is established; the excrementious character of bile is negatived; and the other term of the antithesis follows necessarily from its exclusion.

It is a matter of some regret, that in observations upon dogs affected with biliary fistulæ, attention was not extended to the urine also; but the data furnished by experiments, directed originally to a different end, are entirely competent to decide the point now in question.

By scanning the results of Lehman's series of experiments upon himself, with a view of determinimg the relative constituents of urine under various kinds of diet, we learn that upon a purely nitrogenous or animal diet, he excreted in 24 hrs. 53.2 grm. urea; upon a purely *non* nitrogenous diet, 15.4 grm. urea in the same time. This last, then, may finally be assumed to represent the normal daily amount contributed from all sources in the body, independently of food—from secretions and disassimilative process. Now urea bears to the solids of urine the proportion .524; hence 15.4 grm urea represent 29.6 gram. solid matters in the the urine in 24 hrs.; and since the amount of bile secreted in 24 hrs. is known to be affected to a scarcely perceptible degree by various foods and conditions, its solid matters in the same time would aggregate 30 grm.—i. e., the solid matters of bile secreted exceed the solid matters of urine in the given time by .4 grm.

But beyond doubt much of the urea is the product of disassimilation of nitrogenized tissues; so that the real excess of bile solids secreted over the portion by any possibility excreted in urine must be very large indeed—if in fact any is so secreted.

Some additional weight is given to this by the experiments of Prof. A. Flint, Jr., upon the pedestrian Weston, and corroborated by the observations of Dr. Pavy upon the same individual. These gentlemen found that urea—the element of urine, which must contain the major portion of bile solids excreted by this route—is dependent in amount upon muscular exercise more than any or all causes else.

During the five days' walk, included in the period of observation, Weston ingested daily in food 234.76 gr. nitrogen. The entire daily amount excreted in urine and fæces was 361.52 gr. Now, 234.76 gr. must represent all the nitrogen by any possibility derived from food ingested. Hence, 361.52 gr.—234.76= 126.76 gr. must represent the portion of nitrogen furnished from all sources in the body independently of food—*i. e.*, from disintegrated tissues and bile, say, five times this, or 633.8 gr., equal the amount from these sources during the day's walk.

In the same period, the solid matters of bile alone would equal 2,310 gr., and by quantitative analysis of its nitrogenized constituents, the nitrogen thus supplied would be 34,175 gr. Now, the loss of weight actually sustained during the walk was 26072 gr., 3 per cent. of which, or 782.2 gr., were nitrogen. That is, the nitrogen of the disintegrated tissues alone exceeded, by 148.4 gr., the entire amount excreted by both fæces and urine, over and above that derived from food. The products of disassimilation *must* be excreted, so that it is difficult to see how bile could have furnished any part of the amount. The conclusion seems irresistible, therefore, that but a small portion, if any, of the bile solids or fluids escape from the body through the way of the urine.

An interesting question presents itself here as to the ultimate destination of bile. It is constantly secreted and discharged into the duodenum during the process of digestion; it is not excreted as a whole, nor yet appreciably in its elements—what becomes of it?

Most probably it is absorbed from the intestine after performing its part in digestion (if any), and returned to the liver, to be again secreted and discharged—a sort of circulation of bile similar to that of blood.

Lafter injected the coloring matter of rhubarb and sulphate of soda into the duodenum of a dog, and observed in the space of a minute the same colors in the bile collected from a biliary fistula.

Schiff injected 180 c. c. fresh ox bile into the duodenum of a dog that, previous to the experiment, had discharged 2 c. c. bile from his fistula in 20 minutes. After waiting a short while, the amount collected in 20 minutes from the fistula was 10 c. c., and at the end of an hour after the injection 8.5 c. c. in 20 minutes.

Further, the experiments of Heidenhain show that bile of guinea-pigs, as a rule, gives no reaction with Pettenkoffer's test; yet, if ox bile be injected into the duodenum of one affected with a biliary fistula, in one-half hour thereafter, and for one, two, three hours, a distinct reaction is given by the bile collected from the fistula. This gentleman also observed a change in the color of dog bile soon after the injection of fresh ox bile into the duodenum.

This would explain a fact generally observed, that the amount of bile secreted in a given time is diminished by purgation; where doubtless the bile is swept out of the intestine, entangled in its contents, ere it can be absorbed. Thus the amount secreted by the liver is diminished, until this unexpected loss can be repaired from the resources at hand. This is precisely similar to the fact observed by Schiff, that the rate of secretion is at once diminished by diversion of bile from the intestine.

This concludes what we have to say upon the excrementitious character of bile. We have shown that neither is it, or any considerable portion of its elements, excreted by the urine and fæces severally, nor collectively by both.

(2) Is there now any *positive* evidence that bile is intimately concerned in digestion?

At the outset here, it is a significant fact that ingestion of food is followed immediately, and for some time thereafter, by a largely increased flow of bile into the intestine—a fact more marked, it is true, in animals that feed less frequently than man; yet in man sufficintly marked to indicate an epoch in its flow. Furthermore, after reaching its climax, it gradually subsides, as the digestive process approaches completion, into a condition of stasis until the stimulus is reversed. And if this be indefinitely withheld, establishing the condition of fasting, and the animal killed in this state, the bile bladder is invariably found to be distended with the secretion; whereas, if killed soon after feeding, the bladder is always found empty. I shall not dwell at any length upon the class of sensations, referable to digestion, which by common consent is attributed to disordered function of the liver. This vulgar opinion may not possess great scientific significance of itself; but certain it is, that such symptoms are most generally relieved by administration of those agents which act electively upon the liver, and stimulate it to greater activity.

In further corroboration of this view is the observation of Eaglesfield Smith, that great benefit accrued to a patient affected with jaundice from the administration of fresh bile from a mammal. It is not unworthy of remark, too, at this juncture, the close analogy subsisting between the symptoms characteristic of a well-developed case of jaundice, and those exhibited by animals affected with biliary fistulæ. The same fetid fæces and flatus, the same progressive emaciation characterize both; and if in the former the condition be not arrested, the same result ensues in both; a correspondence which might have been anticipated, since the result should be the same, whether bile be diverted from the digestive process directly by biliary fistula, by occlusion of the ductus communis or by refusal of blood to surrender in the liver its biliary material. The result is in reality the same—absence of bile from the digestive process; and in all cases alike nutrition is interrupted.

We come now to the consideration of a class of experiments, to which objection has been made, rather impertinently, that they establish a pathological rather than a physiological condition. So they do; but it is this very pathological condition, which remains and gradually increases in gravity after the immediate effects of the operation have subsided; it is this very condition that is fraught with information in reference to the function of bile in digestion. For, if it performed no function, then should its absence from the process be attended by no untoward consequences. We refer to experiments upon animals in which the bile was diverted from the intestine by means of biliary fistulæ. And first in reference to perseverance, success and uniformity of results, stands Schwan.

He established biliary fistulæ in eighteen dogs, ten of which died from the direct effects of the operation. In two the duct was re-established, and the remaining six, in which the bile was completely diverted, all died in 7, 13, 17, 25, 64 and 80 days, respectively, after the operation, with all the symptoms of profoundly disturbed nutrition—inanition, loss of flesh and muscular power, unsteadiness of gait, falling of hair.

Subsequent experiment upon 30 dogs by the same observer, corroborated his former results. All died; though one lived four months, and one a year—the symptoms gradually increasing in gravity until death ensued.

Precisely similar in all essential particulars were the results obtained by Bidder and Schmidt from similar experiments. The several subjects of their observation, as well as Claude Bernard's, lost weight steadily, though appetite was unimpaired, and even increased in voracity; the skin became loose; muscles were scarcely perceptible; hair fell out. They all noted the exceedingly disgusting odor of the fæces and breath, and abundance of flatus in the intestine.

Prof. A. Flint, Jr., also performed, with distinguished success, the operation establishing a biliary fistula upon a dog, completely occluding the common duct and diverting the bile. This dog passed through all the stages above described, dying at length on the 38th day after the operation. The observer noted no fetor of breath in this case; but adds that, though the animal ate ravenously of lean meats, it could not be induced to touch fats, even after fasting a considerable time. In all these cases the occlusion of the ductus communis was demonstrated by post-mortem dissection.

Nasse claims to have kept a dog affected with a biliary fistula alive for five months without appreciable loss of weight; but it died at the end of that time without any apparent cause. The general results, however, obtained by this observer corroborate those given above.

As an offset to these numerous cases, we have now to consider two individual cases of an apparently contradictory character. One is furnished by Bloudlot, whose dog was allowed at first to lick the bile as it flowed from the fistula, but was afterward prevented by a muzzle. It lived five years in good health, being used meantime for hunting. Post-mortem dissection in presence of several physicians and students showed the duct to be entirely obliterated. It is to be remembered, however, in this case, that except during the first and last three months of the five years, the dog was not under Bloudlot's observation at all; and we are not informed whether it was allowed in the interim to lick the bile as at first, or even whether the fistula remained open. So that, whatever contradictory weight this might have, must be discounted in the absence of information upon these points.

The other is supplied from the reports of the Edinburgh Committee, under whose observation, as reported, a dog similarly affected was kept five months, with a gain meantime of four kilogm. in weight. It is by no means certain, however, that in both these cases a duct was not improvised under the conservative laws of nature; or that, under the same law, sufficient bile did not find its way into the intestine by osmosis. More remarkable provisions than either of these supposed have occurred in the animal economy. And the former explanation gains credence from an experiment related by Prof. Flint, in which the dog lost the canula from his fistula. The wound soon healed, and some months afterward the dog, apparently in good health, was killed. Upon examination the ductus communis *seemed* to be obliterated in this case also, and was in reality; but a tedious and careful dissection of neighboring tissues was at length rewarded by the discovery of a small, tortuous canal, through which bile found its way into the intestine. Like perseverance in the cases above cited might, and would in all probability, have discovered a similar provision. But discounted though the numerous concurrent cases be by these isolated experiments; yet, in view of all the evidence, there can be no rational doubt that bile performs some important function in the process of digestion. Indeed, this seems to be as well established as any other general physiological fact.

As this brings us to the conclusion of the first division of the subject, let us summarize the points established: (1) Bile is not excreted to any considerable extent, either (a) with the fæces, or (b) with the urine; and even the small portion thus excreted is most probably carried out by accidental entanglement with excretory principles. But there seems to be rather a circulation of bile, in some respects similar to that of blood. (2) By sequence from (1) and by disturbance of nutrition exhibited in its absence, it must enter as an important factor in digestion.

II. What then is that function which it performs? That its function should be complex and not clearly marked like that of gastric juice, might, as we have stated above, have been anticipated *a priori* from its very composition—comprising a variety of complex substances more or less loosely united, most of which are readily resolved into their ultimate elements, possessing distinctive reactions. Moreover, we do know that such resolution transpires with more or less facility in various parts of the intestinal canal.

(1) First, then, bile seems to aid in digestion by retarding putrefaction, which is prone to occur when a variety of disintegrated and disintegrating substances are collected together in the presence of heat. This is attested by the unbroken testimony of experimenters to the offensive odor of fæces, when bile is diverted from digestion, and to the presence of flatus in large quantities under similar circumstances—both of which are invariable concomitants of putrefaction within and without the intestine.

It is established by experimentation extra-intestinal. Von Gorup Besauez demonstrated that, when meat is digested with bile the temperature of the body, the putrefaction process is postponed for a considerable period beyond that at which it is set up under the same circucumstances without bile.

A similar postponement of fermentation occurs in syrup and urine, according to the same observer. Moreover, Claude Bernard found that fermentation in yeast taken from a dog's stomach was completely arrested in presence of bile.

Stolukoff gives the following comprehensive and intelligent variations of these experiments. Bile and water; fibrin, bile, water; fibrin, fat, water; fibrin, fat bile, water; were grouped as above in separate bottles, and allowed to remain undisturbed from the middle of June to the middle of August. The gas, collected from each over mercury, was, at the end of the time, found to be greatest in quantity from the jar containing fat and fibrin without bile. Though the water and bile had split up into new products, there was no gas given off from the jar containing them alone. The gas collected showed about 92 per cent. $CO_2$; while the bile acids had split up into glycocol, taurin and cholalic acid.

Bidder and Schmidt also kept meat and albumen 48 hours in solution of bile acids at the temperature of the body, without at the end of that time any putrefactive changes being apparent; whereas the same substances treated simply with water gave distinct signs of such changes in 24 hours. But it is not necessary to further multiply testimony on this point.

(2) Bile or some of the products of its decomposition acts as a persuader of peristalsis in the bowel. This property seems to be enjoyed by its pigments and its acids; but in an especial degree by cholalic acid—as shown on the one hand by Shülein, who found purging and vomiting follow the administration of bile acids; and on the other by the statements of Tiedman and Gruelin, and Eberle, to the effect that all dogs with biliary fistulæ under their observation severally were costive, and the fæces very hard and white. Bile seems, therefore to be a natural inviter of peristaltic action and intestinal secretion.

(3) Bile emulsifies fats to a certain extent, and facilities their absorption. This seems to be the most generally conceded of all its properties; and has been attributed to it since the earliest times. The former is easily demonstrated in a test-tube; the latter by the fact, first demonstrated by Von Wittinghausen, that fats find their way through animal membranes or plaster wetted with bile and at the temperature of the body, much more readily than when dry or moistened with water—indeed there is no evidence that fats are ever absorbed in any marked degree, save through the action of bile upon the membranes of the walls of the intestines. How this is effected does not appear. Most probably the fats are subjected to finer subdivision or else the stomata of the membrane are modified thereby—or possibly both these occur.

The absence of fat through failure of these modifications would account for the clear, colorless character of chyle observed by Brodie, Bidder and Schmidt, Magendie, and Tiedman and Gmelin, in dogs with biliary fistulæ or tied ducts. According to these last two, chyle of dogs in normal condition was white and milky, while the liquid found in both lacteals and thoracic ducts of those with tied ducts was clear and translucent, the difference being due to lack of finely divided particles of fat in the latter.

However it may effect its purpose, one fact is incontrovertible, namely, that the presence of good healthy bile is a *sine qua non* to the elaboration of fats in the animal economy.

(4.) Fresh human bile, in common with that of many mammals, in a limited extent converts starch and hepatic glycogen into sugar. (Bufalini and Whittish.)

It possesses no power over the sugars themselves. (Frerichs and Shiel.)

Whether this is an original and invariable property, it is impossible to say; but it is difficult to see how so limited an action could be necessary to vitality. If, however, sugar in certain proportion be a necessity to the healthy performance of vital functions, it is conceivable that, in certain conditions where the direct sources of sugar supply are cut off, this power of bile over hepatic glycogen might rise to considerable prominence.

These in the aggregate constitute the function of bile in digestion, so far as can be predicated with any certainty in the premises. Are they sufficient to account for the presence of bile in the intestine? We can only say in reply, that the absence of bile from the intestine is invariably attended by an almost total suspension of these actions—several or all of them—and the suspension of these actions for any considerable period produces death. For our part, we cannot see why elaboration of fats, repression of putrefaction, and promotion of peristalsis, should not collectively constitute a satisfactory account of its presence—certainly they are essential to existence.—*New Orleans Medical and Surgical Journal.*

